# Perceived knowledge, attitude, and practice of artificial intelligence among medical students in Guangxi: a cross-sectional study

**DOI:** 10.3389/fpubh.2026.1824962

**Published:** 2026-06-02

**Authors:** Lulin Chen, Wei Liu, Yanting Zhou

**Affiliations:** 1 Department of Scientific Research, The Second Nanning People’s Hospital, Nanning, China; 2 School of Foreign Languages, Guangxi University of Science and Technology, Liuzhou, China; 3 Academic Affairs Office, Guangxi Medical University, Nanning, China

**Keywords:** artificial intelligence, attitude, knowledge, medical education, medical student, practice

## Abstract

**Introduction:**

This study aimed to explore the perceived knowledge, attitude, and practice (KAP) regarding artificial intelligence (AI) of medical students in Guangxi, China.

**Methods:**

A cross-sectional survey was conducted from October to November 2024 at two universities in Guangxi, China. The survey assessed students’ KAP regarding AI using a 5-point Likert scale. Quantile regression models (*τ* = 0.5) were used to identify factors associated with KAP scores.

**Results:**

A total of 894 undergraduate medical students were enrolled. Participants demonstrated a moderate level of perceived AI knowledge (mean score 13.36 ± 3.26) and a moderately positive attitude (mean score 38.49 ± 5.69). However, AI practice was low (mean score 15.40 ± 4.57), with the highest frequency of AI practice for exam preparation (mean score 2.40 ± 0.84). Gender, academic year, major, hometown, visiting a science museum or exhibition in the past year, and studying AI during undergraduate education were associated with perceived AI knowledge. Gender, visiting a science museum or exhibition in the past year, and perceived AI knowledge were associated with attitude towards AI. Gender, academic year, studying AI during undergraduate education, perceived AI knowledge, and attitude towards AI were associated with AI practice. Significant barriers included limited practical opportunities, lack of specialized textbooks and courses, and insufficient professional guidance.

**Conclusion:**

Medical students in Guangxi have moderate perceived AI knowledge and positive attitudes, but structural barriers (limited practical opportunities, lack of specialized textbooks/courses, and insufficient professional guidance) hinder AI integration into medical education. Based on these findings, we propose: integrating elective AI modules with hands-on workshops using open-source tools; developing open-access, low-cost learning materials and faculty training for rural-serving institutions; and fostering cross-disciplinary collaborations to apply AI to clinical cases. Future research should evaluate such interventions and address structural inequalities in AI learning.

## Introduction

Artificial intelligence (AI) has rapidly emerged as a transformative technology with significant potential to revolutionize healthcare (1). Its applications in medicine, including diagnostic support, personalized treatment plans, and medical imaging, have demonstrated the capacity to enhance clinical outcomes and improve patient care (1, 2). As AI continues to evolve, its integration into medical education becomes increasingly important to equip future healthcare professionals with the necessary skills and knowledge to navigate the evolving healthcare landscape (3, 4). However, despite the growing relevance of AI in medicine, its incorporation into medical curricula remains limited, particularly in regions such as Asia, where research on this topic is still emerging (5–7).

Medical students’ knowledge, attitude, and practice (KAP) regarding AI are critical factors influencing the successful integration of this technology into medical education and future practice (8). While some studies have highlighted the potential benefits of AI in healthcare, others have identified significant gaps in students’ understanding of AI, skepticism about its impact on traditional medical roles, and concerns regarding ethical implications and technical complexity (9, 10). These mixed perceptions underscore the need to better understand the KAP regarding AI among medical students (8). Furthermore, identifying barriers to AI integration, such as lack of access to technical equipment, limited educational exposure, and time constraints, is essential to inform strategies that promote effective AI adoption in medical education (1).

Although several studies have explored the KAP of medical students regarding AI in China (11, 12), these have primarily been conducted in more developed eastern regions (13–15). Similar investigations remain scarce in less developed regions, such as Guangxi. As a region with a developing economy and a significant proportion of its population residing in rural areas, students in Guangxi may face unique challenges in accessing AI education and resources, such as disparities in technological infrastructure and educational exposure (16, 17). Given the growing importance of AI in healthcare and the need for equitable educational development, this study aimed to explore the KAP regarding AI among medical students in Guangxi. By examining their perspectives on AI education and identifying factors influencing their AI practice, this research seeks to provide insights that can inform the development of targeted educational interventions and support the integration of AI into medical curricula.

## Methods

### Study design

A cross-sectional survey was conducted from October 2024 to November 2024 at two universities in Guangxi, China, with a target population of approximately 5,316 undergraduate medical students. Convenience sampling was applied. The Chinese-language questionnaire and study invitation were distributed electronically to eligible students through mobile application platforms by clinical professional teaching administrators. The introductory information emphasized that participation was voluntary and that the collected data would be kept confidential and used solely for research purposes. Both the informed consent form and the questionnaire were completed online. After reading and submitting the informed consent form, participants had the option to fill out the questionnaire, which was then returned anonymously. Ethical approval was obtained from the Institutional Review Board.

### Data collection and study instruments

The questionnaire was structured according to the KAP model, a widely used theoretical framework in health education and behavioral research. The KAP model posits that knowledge (understanding of a topic) shapes attitude (beliefs and feelings toward that topic), which in turn influences practice (actual behaviors or actions). This causal chain has been applied to investigate healthcare professionals’ adoption of new technologies, including AI in medicine (18). The KAP questionnaire was newly developed for this study. The research team first drafted the initial items in English based on a systematic literature review of AI in medical education and by referencing the content and structure of previous KAP studies (14, 19–23) without directly adapting or replicating any single existing tool. The initial English-language items underwent cross-cultural adaptation, including forward translation into Chinese and independent back-translation to ensure conceptual equivalence. The questionnaire comprised five main parts: (1) demographics, (2) perceived AI knowledge (5 items), (3) AI attitude (12 items), (4) AI practice (7 items), and (5) perspectives on AI learning (5 items).

Scoring for KAP used a 5-point Likert scale ranging from “completely do not know” (1 point) to “completely know” (5 points) for perceived knowledge, from “strongly disagree” (1 point) to “strongly agree” (5 points) for attitude, and from “never” (1 point) to “all the time” (5 points) for practice, with total scores of 25, 60, and 35, respectively. The perspective section investigated perceived learning requirements, preferred learning methods, perceived learning content, encountered difficulties, and barriers to AI application (14, 21–23). Participants were provided with a list of relevant options.

### Instrument validation

To ensure the validity and reliability of the questionnaire, an expert panel comprising four professors from clinical medicine, medical imaging, preventive medicine, and educational management was convened to evaluate its content validity. The four experts independently rated each item on relevance and representativeness of the intended domain using a 5-point Likert scale, ranging from “completely not relevant/representative” (1 point) to “completely relevant/representative” (5 points). The item-level content validity index (I-CVI) for relevance and representativeness was calculated separately as the proportion of experts assigning a rating of 4 or 5 for each criterion. Items with I-CVI below 0.75 on either relevance or representativeness, or those with major wording suggestions from at least two experts, were revised or removed. The detailed expert ratings and the resulting item-level content validity indices are provided in Supplementary Table S1.

A pilot study involving 30 students (excluded from the final analysis) was conducted to verify the clarity of each question. The average completion time established in the pilot was approximately 5 min. Internal consistency of the KAP questionnaire was assessed using Cronbach’s *α* coefficient and the Kaiser-Meyer-Olkin (KMO) index. For the perceived knowledge subscale (5 items), Cronbach’s *α* = 0.890 and KMO = 0.852. For the attitude subscale (12 items), *α* = 0.852 and KMO = 0.900. For the practice subscale (7 items), α = 0.906 and KMO = 0.893. For the total 24-item KAP scale, Cronbach’s α = 0.882 and KMO = 0.897, indicating high overall reliability and excellent factorability.

We computed corrected item-total correlations (CITC) for each subscale using the pilot sample (Supplementary Table S2). The perceived knowledge subscale had a CITC range of 0.43–0.48; the attitude subscale ranged from 0.14 to 0.57; and the practice subscale ranged from 0.42 to 0.56. Most items exhibited satisfactory CITC values (>0.30), providing preliminary evidence of construct validity. However, three attitude items showed lower correlations: item (“human teachers will be replaced”) had CITC = 0.26, item (“Clinical AI will be more accurate than physicians”) had CITC = 0.29, and item (“AI would increase diagnostic errors”) had CITC = 0.14. These three items also had low factor loadings in the exploratory factor analysis (EFA) (see below). Despite their suboptimal psychometric properties, we retained them because they capture distinct negative perceptions of AI (e.g., concerns about replacement, accuracy, and safety) that are theoretically important for understanding medical students’ full range of attitudes. Excluding these items would have raised the scale’s *α* slightly (to 0.89), but the gain was considered insufficient to justify removing meaningful content.

To empirically confirm the three-dimensional structure (perceived knowledge, attitude, practice), we performed an EFA using principal axis factoring with varimax rotation on the 24 KAP items. The Bartlett’s test of sphericity was significant (*p* < 0.001). A three-factor solution emerged, explaining 55.29% of the total variance. As shown in the rotated component matrix (Supplementary Table S3), 21 of the 24 items had primary loadings ≥ 0.50 on their expected subscale (perceived knowledge: Factor 3, loadings 0.77–0.85; attitude: Factor 1, loadings 0.57–0.81; practice: Factor 2, loadings 0.69–0.85). However, three attitude items (“human teachers will be replaced,” “Clinical AI will be more accurate than physicians,” “AI would increase diagnostic errors”) exhibited consistently low loadings (<0.30) on all three factors. The factor structure of the remaining 21 items supported the three-dimensional KAP framework.

### Inclusion and exclusion criteria

Eligible participants were undergraduate medical students enrolled at the participating institutions. We excluded incomplete questionnaires and those with a response time <5 min. This threshold was based on the pilot study, where the average completion time was 5 min. We set the exclusion threshold to remove responses that were likely to be inattentive or random.

### Sample size calculations

The minimum sample size was determined using the Morgan equation (24), with a 95% confidence level and a 5% margin of error, resulting in a requirement of 385 participants. The study ultimately enrolled 894 students.

### Statistical analysis

Data were analyzed using SPSS version 27.0. Categorical variables were summarized using frequencies and percentages, while continuous variables were expressed as the mean with standard deviation. To identify factors associated with KAP scores, three quantile regression models were fitted at the conditional median (*τ* = 0.5). The median was selected because it is robust to non-normal distributions and outliers, aligning with the primary justification for using quantile regression. Moreover, our research objective was to estimate the effects of covariates on the central tendency of KAP scores, rather than on the lower or upper tails (25). The perceived knowledge model incorporated academic year, gender, hometown, major, visiting a science museum or exhibition, and studying AI during undergraduate education as independent variables. The attitude model included these same variables plus the perceived knowledge score. Similarly, the practice model added both perceived knowledge and attitude scores to the independent variables. A *p*-value of less than 0.05 was considered statistically significant.

## Results

A total of 973 questionnaires were initially submitted. After applying the exclusion criteria, 79 invalid responses were excluded. This study ultimately enrolled 894 participants (56.7% female and 43.3% male). Most participants (82.7%) were in academic year 5, because they had more frequent access to the mobile distribution platform through clinical teaching administrators during their internship rotations. The participants were predominantly students of clinical medicine and general practice, accounting for 75.2% of the total. Six hundred nineteen participants (69.2%) came from rural hometowns. Six hundred thirty-four participants (70.9%) had not visited a science museum or exhibition in the past year, and 593 participants (66.3%) had not studied AI during their undergraduate education. Table 1 presents the participants’ characteristics.

**Table 1:** Participants’ characteristics.

Variables	*n* (%)
Academic year	
1	3 (0.3)
2	17 (1.9)
3	69 (7.7)
4	66 (7.4)
5	739 (82.7)
Gender	
Female	507 (56.7)
Male	387 (43.3)
Major	
Clinical Medicine	507 (56.7)
General Practice	165 (18.5)
Pediatrics	70 (7.8)
Anesthesiology	20 (2.2)
Medical Imaging	26 (2.9)
Psychiatry	35 (3.9)
Others	71 (7.9)
Hometown	
Rural	619 (69.2)
Urban	275 (30.8)
Have you visited a science museum or exhibition in the past year?	
Yes	260 (29.1)
No	634 (70.9)
Did you study artificial intelligence during your undergraduate education?	
Yes	301 (33.7)
No	593 (66.3)

Participants demonstrated a moderate level of perceived AI knowledge. The majority of respondents fell into the “Know some” category for most items (ranging from 44.5 to 57.2%). The mean total score was 13.36 ± 3.26. The item “AI requires a lot of labeled data to learn (data already processed by a human)” had the highest mean score (2.84 ± 0.89), and the item “Do you know what deep learning/machine learning is?” had the lowest mean score (2.50 ± 0.77). Perceived AI knowledge of medical students is shown in Table 2.

**Table 2:** Perceived knowledge of medical students regarding AI [*n* (%)].

Items	Completely know	Know	Know some	Know a little	Completely do not know	M ± SD
Do you have a solid knowledge of the basics of AI?	6 (0.7)	59 (6.6)	511 (57.2)	254 (28.4)	64 (7.2)	2.65 ± 0.74
Do you know what deep learning/machine learning is?	7 (0.8)	53 (5.9)	398 (44.5)	356 (39.8)	80 (8.9)	2.50 ± 0.77
Do you know any application of AI in your field of interest?	6 (0.7)	84 (9.4)	511 (57.2)	237 (26.5)	56 (6.3)	2.72 ± 0.75
AI requires a lot of labeled data to learn (data already processed by a human)	18 (2.0)	180 (20.1)	399 (44.6)	236 (26.4)	61 (6.8)	2.84 ± 0.89
I understand the barriers to applying AI in medicine	9 (1.0)	71 (7.9)	466 (52.1)	293 (32.8)	55 (6.2)	2.65 ± 0.76
Total score	–	–	–	–	–	13.36 ± 3.26

Medical students demonstrated a moderately positive attitude towards AI, with the three highest mean scores observed for “I believe ethical implications of AI must be understood by all medical students” (3.62 ± 0.86), “I believe AI would promote medical development” (3.61 ± 0.72), and “I believe AI will be an essential tool in my field” (3.50 ± 0.82). Conversely, the three lowest mean scores were for “I believe human teachers will be replaced in the foreseeable future” (2.41 ± 0.92), “I believe clinical AI will be more accurate than physicians” (2.62 ± 0.80), and “I believe AI would increase the percentage of errors in diagnosis” (3.00 ± 0.62). The mean total score was 38.49 ± 5.69, reflecting an overall moderate attitude towards AI. Table 3 presents the medical students’ attitude towards AI.

**Table 3:** Medical students’ attitude towards AI [*n* (%)].

Items	Strongly agree	Agree	Neutral	Disagree	Strongly disagree	M ± SD
I believe healthcare students should learn the basics of AI	36 (4.0)	150 (16.8)	578 (64.7)	101 (11.3)	29 (3.2)	3.07 ± 0.75
I believe AI will be an essential tool in my field	88 (9.8)	351 (39.3)	394 (44.1)	43 (4.8)	18 (2.0)	3.50 ± 0.82
I believe the ethical implications of AI must be understood by all medical students	136 (15.2)	354 (39.6)	350 (39.1)	37 (4.1)	17 (1.9)	3.62 ± 0.86
I believe AI will revolutionize the educational system	43 (4.8)	210 (23.5)	516 (57.7)	99 (11.1)	26 (2.9)	3.16 ± 0.79
I believe human teachers will be replaced in the foreseeable future	16 (1.8)	57 (6.4)	367 (41.1)	293 (32.8)	161 (18.0)	2.41 ± 0.92
I believe the upcoming developments in the educational system will excite me	40 (4.5)	310 (34.7)	495 (55.4)	30 (3.4)	19 (2.1)	3.36 ± 0.72
I believe AI should be a part of the training system for medical students	42 (4.7)	372 (41.6)	436 (48.8)	32 (3.6)	12 (1.3)	3.45 ± 0.70
I believe clinical AI will be more accurate than physicians	12 (1.3)	59 (6.6)	483 (54.0)	256 (28.6)	83 (9.3)	2.62 ± 0.80
I believe some specialties are more prone to be replaced by AI than others	39 (4.4)	327 (36.6)	452 (50.6)	58 (6.5)	17 (1.9)	3.35 ± 0.75
I believe AI would increase the percentage of errors in diagnosis	13 (1.5)	114 (12.8)	642 (71.8)	109 (12.2)	16 (1.8)	3.00 ± 0.62
I believe AI should be integrated into the medical curriculum	47 (5.3)	293 (32.8)	482 (53.9)	54 (6.0)	18 (2.0)	3.33 ± 0.75
I believe AI would promote medical development	73 (8.2)	443 (49.6)	345 (38.6)	24 (2.7)	9 (1.0)	3.61 ± 0.72
Total score	–	–	–	–	–	38.49 ± 5.69

Medical students reported relatively low frequencies of AI practice across various activities, with the highest mean score for using AI to prepare for exams (2.40 ± 0.84). Conversely, the lowest mean score (2.01 ± 0.80) was reported for using AI for personal choices and career guidance. The total score for AI practice across all activities was 15.40 ± 4.57. Table 4 presents the medical students’ AI practice.

**Table 4:** Medical students’ AI practice [*n* (%)].

Items	All the time	Most of the time	Often	Rarely	Never	M ± SD
How frequently do you use AI to prepare for your exams?	16 (1.8)	54 (6.0)	311 (34.8)	405 (45.3)	108 (12.1)	2.40 ± 0.84
How frequently do you use AI to prepare for your homework/assignments?	9 (1.0)	45 (5.0)	329 (36.8)	416 (46.5)	95 (10.6)	2.39 ± 0.78
How frequently do you use AI to conduct your research?	6 (0.7)	35 (3.9)	218 (24.4)	423 (47.3)	212 (23.7)	2.11 ± 0.83
How frequently do you use AI for idea generation and brainstorming?	7 (0.8)	33 (3.7)	201 (22.5)	449 (50.2)	204 (22.8)	2.09 ± 0.81
How frequently do you use AI for personal choices/career guidance?	8 (0.9)	24 (2.7)	172 (19.2)	452 (50.6)	238 (26.6)	2.01 ± 0.80
How frequently do you use AI for spelling and grammar checking?	14 (1.6)	39 (4.4)	245 (27.4)	441 (49.3)	155 (17.3)	2.23 ± 0.84
How frequently do you use AI for personality development and other skills?	11 (1.2)	28 (3.1)	221 (24.7)	471 (52.7)	163 (18.2)	2.16 ± 0.80
Total score	–	–	–	–	–	15.40 ± 4.57

**Figure 1:**
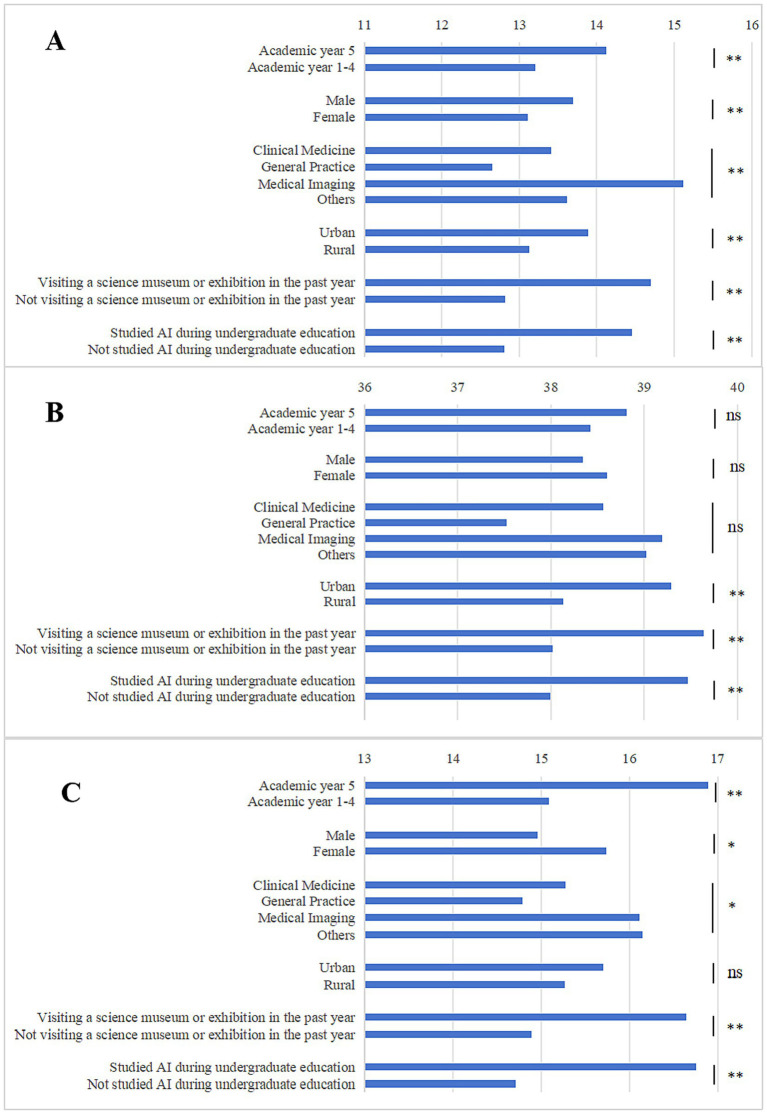
Comparison of KAP scores across different demographic subgroups. **(A)** Perceived knowledge scores; **(B)** attitude scores; **(C)** practice scores. Group differences were evaluated using *t*-tests (for two-level variables) or one-way ANOVA (for multi-level variables). Significance levels: *p < 0.05, **p < 0.01 (two-tailed).

As depicted in Figure 1A, the total perceived knowledge score varied among different subgroups (*p* < 0.01). In contrast, the overall attitude towards AI demonstrated considerable consistency (Figure 1B). Notably, the mean attitude scores showed no statistical differences across academic years, genders, and majors (*p* > 0.05). Figure 1C illustrates the comparison of practice scores across various demographic factors. A key observation is that the frequency of AI practice was similar between students from rural and urban hometowns (*p* > 0.05).

Table 5 presents the results of the quantile regression model (*τ* = 0.5) examining factors associated with medical students’ KAP scores regarding AI. Only variables with statistically significant coefficients (*p* < 0.05) are shown for brevity, and the full regression outputs are provided in Supplementary Tables S4–S6. Male students (coefficient = 1.000, *p* < 0.001; 95% CI: 0.592–1.408), urban background (coefficient = 1.000, *p* < 0.001; 95% CI: 0.557–1.443), a visit to a science museum or exhibition in the past year (coefficient = 1.000, *p* < 0.001; 95% CI: 0.539–1.461), and prior study of AI during undergraduate education (coefficient = 1.000, *p* < 0.001; 95% CI: 0.560–1.440) were associated with higher perceived AI knowledge. Academic years 1–4 were associated with higher perceived AI knowledge compared to academic year 5 (coefficient = 1.000, *p* < 0.001; 95% CI: 0.416–1.584). Clinical medicine, general practice and other majors were associated with lower perceived AI knowledge compared to medical imaging (*p* < 0.01).

**Table 5:** Quantile regression (τ = 0.5) results for factors associated with medical students’ KAP scores.

Variables	Coefficient	SE	*t*	*p*	95% CI
Independent variable: Perceived AI knowledge					
Male	1.000	0.208	4.805	<0.001	0.592–1.408
Urban	1.000	0.226	4.434	<0.001	0.557–1.443
Visited a science museum or exhibition in the past year	1.000	0.235	4.257	<0.001	0.539–1.461
Studied AI during undergraduate education	1.000	0.224	4.459	<0.001	0.560–1.440
Academic year 1–4 ^a^	1.000	0.297	3.362	0.001	0.416–1.584
Clinical medicine ^b^	−2.000	0.618	−3.235	0.001	−3.213--0.787
General practice ^b^	−2.000	0.647	−3.092	0.002	−3.270--0.787
Other majors ^b^	−2.000	0.639	−3.132	0.002	−3.253--0.747
Independent variable: Attitude towards AI					
Male	−0.750	0.366	−2.050	0.041	−1.468--0.032
Visited a science museum or exhibition in the past year	1.250	0.419	2.983	0.003	0.427–2.073
Perceived AI knowledge	0.250	0.058	4.322	<0.001	0.136–0.364
Independent variable: AI practice					
Male	−0.833	0.320	−2.604	0.009	−1.461–0.205
Studied AI during undergraduate education	1.250	0.348	3.595	<0.001	0.568–1.932
Perceived AI knowledge	0.278	0.052	5.319	<0.001	0.175–0.380
Attitude towards AI	0.083	0.029	2.928	0.003	0.027–0.139
Academic year 1–4 ^a^	1.444	0.456	3.171	0.002	0.550–2.339

^a^Taking academic year 5 as a reference. ^b^Taking medical imaging major as a reference.

Male students had a more negative attitude towards AI (coefficient = −0.750, *p* = 0.041; 95% CI: −1.468 to −0.032). Visiting a science museum or exhibition in the past year (coefficient = 1.250, *p* = 0.003; 95% CI: 0.427–2.073), and having higher perceived knowledge of AI (coefficient = 0.250, *p* < 0.001; 95% CI: 0.136–0.364) were associated with a more positive attitude.

Male students had a lower frequency of AI practice (coefficient = −0.833, *p* = 0.009; 95% CI: −1.461 to −0.205). Studying AI during undergraduate education (coefficient = 1.250, *p* = 0.001; 95% CI: 0.568–1.932), being in academic years 1–4 (coefficient = 1.444, *p* = 0.002; 95% CI: 0.550–2.339), having higher perceived AI knowledge (coefficient = 0.278, *p* < 0.001; 95% CI: 0.175–0.380), and holding a more positive attitude towards AI (coefficient = 0.083, *p* = 0.003; 95% CI: 0.027–0.139) were associated with a higher frequency of AI practice.

Table 6 presents medical students’ perspectives on AI education. When asked about learning requirements, the majority believed that basic knowledge in daily life is sufficient (475, 53.1%), while 320 students (35.8%) considered in-depth study necessary. Only a small proportion felt that basic learning (85, 9.5%) or no learning at all (14, 1.6%) would be adequate. Medical students expressed a preference for elective AI courses (545, 61.0%) and highlighted the importance of understanding the intersection of AI and medicine (761, 85.1%), and AI applications in various fields (618, 69.1%). However, they also identified significant barriers, including lack of practical opportunities (724, 81.0%), lack of specialized textbooks and courses (705, 78.9%), and lack of professional teacher guidance (657, 73.5%). The most prominent barriers to AI application were lack of access/technical equipment (679, 76.0%), followed by lack of knowledge and expertise (627, 70.1%), and lack of time due to educational burden (557, 62.3%). Ethical and privacy concerns were the least frequently reported barrier.

**Table 6:** Medical students’ perspectives on AI learning.

Items		*n* (%)
What do you think are the learning requirements for medical students regarding AI?	In-depth study	320 (35.8)
	Basic learning	85 (9.5)
	Knowledge in daily life is sufficient	475 (53.1)
	No need to learn	14 (1.6)
What do you think the learning methods for AI courses should be?	Core courses	226 (25.3)
	Elective courses	545 (61.0)
	Online courses	123 (13.8)
What do you think the current learning content for AI should include?	The concept of AI	396 (44.3)
	The history of AI	300 (33.6)
	Applications of AI in various fields	618 (69.1)
	The intersection of AI and medicine	761 (85.1)
	Operation and research of new technologies	616 (68.9)
	Ethical issues related to AI	496 (55.5)
	Other	11 (1.2)
What difficulties do you currently face in the process of learning AI?	Lack of professional teacher guidance	657 (73.5)
	Lack of specialized textbooks and courses	705 (78.9)
	Lack of practical opportunities	724 (81.0)
	Lack of motivation to learn	343 (38.4)
	Other	6 (0.7)
What do you think are the barriers to the application of AI?	Lack of knowledge and expertise	627 (70.1)
	Lack of access/technical equipment	679 (76.0)
	Ethical and privacy concerns	441 (49.3)
	Lack of time due to educational burden	557 (62.3)
	Complexity of AI	504 (56.4)
	Limited integration in educational curricula	447 (50.0)
	Lack of teaching centers and hands-on applications	449 (50.2)
	Other	5 (0.6)

## Discussion

This study provides a comprehensive overview of medical students’ KAP regarding AI in Guangxi. The results indicate that students’ perceived AI knowledge is moderate, with significant variations based on gender, hometown, and exposure to AI-related activities. While students hold moderately positive attitudes towards AI, concerns regarding its accuracy and potential impact on traditional medical roles remain. AI practice among students is relatively low, highlighting the need for enhanced educational opportunities and practical exposure. The study also identified key barriers to AI learning and application, including limited practical opportunities and lack of specialized resources.

The study reveals that medical students in Guangxi possess a moderate level of perceived AI knowledge, with the majority falling into the “Know some” category. This finding aligns with previous research from other regions (6, 26, 27). Notably, male students tended to have higher perceived knowledge scores, consistent with previous studies (28, 29). Urban students, those who had visited a science museum or exhibition, and those who had studied AI during their undergraduate education demonstrated higher perceived knowledge scores. This may be attributed to increased exposure and access to AI-related resources in these groups (27). Students majoring in clinical medicine and general practice exhibited lower perceived AI knowledge compared to those in medical imaging. Medical imaging is a field where AI applications are more prevalent and well-integrated into the curriculum and clinical practice (30). This highlights the importance of exposure to AI through coursework and practical experience in shaping students’ knowledge (31).

Medical students hold moderately positive attitudes towards the integration of AI in medical education and healthcare. This optimism is driven by the potential benefits of AI, such as improving diagnostic accuracy, optimizing study time, and providing up-to-date medical information (27). These findings align with studies from other regions, such as Oman and Spain (32, 33). A recent study in Pakistan found that 80.3% of medical students viewed AI favorably, considering it an effective (60.8%) and credible (58.4%) learning tool. Similarly, a Canadian study reported that 94% of medical students believed AI applications in medicine would become more widespread in the future (34). However, our study found that concerns about the potential negative impacts of AI were less pronounced compared to those reported in other regions, where significant proportions of medical students feared that AI could replace human medical professionals (27, 35). In our study, the majority of students did not share these concerns, suggesting a more nuanced understanding of AI’s role in healthcare. Our findings indicate that medical students generally view AI as a valuable tool that can enhance medical education and practice, aligning with global trends where AI is seen as a transformative force in healthcare (36).

Medical students’ AI practice is relatively low, with the highest frequency reported for exam preparation. It is possible that students perceive AI mainly as a study aid (27, 37). The low frequency of AI practice in areas such as personal choices and career guidance indicates that AI is not yet fully integrated into medical education (38, 39). Lower usage in these areas indicates a need for more comprehensive AI integration in educational curricula. This gap is consistent with findings from other studies, which highlight the need for more comprehensive AI training programs to equip students with the skills to effectively use AI in clinical practice (40). Integrating AI into medical education beyond exam preparation could help students better understand its diverse applications. This highlights the importance of educational exposure and positive reinforcement in promoting AI adoption among medical students. Male students were positively associated with higher perceived knowledge scores, but exhibited more negative attitudes and lower AI practice. This finding is consistent with broader research indicating that while students recognize the potential benefits of AI in healthcare, they often lack the practical skills and confidence to integrate it into their clinical practice (18). Additionally, higher perceived AI knowledge and positive attitudes were associated with increased AI practice, reinforcing the importance of educational interventions in promoting AI adoption (18, 26).

Students’ views on AI education reveal a preference for elective AI courses and an emphasis on understanding the intersection of AI and medicine. Students emphasize the importance of understanding how AI intersects with medicine, particularly in areas such as diagnostics, treatment planning, and patient care. A study assessing AI literacy among medical students in Zambia found that students recognized key benefits of AI in healthcare, such as improved diagnostic accuracy and enhanced treatment planning (41). However, they also expressed concerns about potential biases in AI algorithms and the need for ethical considerations in AI integration. This underscores the importance of developing curricula that not only teach technical skills but also address the ethical and practical implications of AI in healthcare. Several barriers to AI learning were identified, including a lack of practical opportunities, specialized textbooks and courses, and professional guidance. These challenges are particularly pronounced in resource-limited settings, where technological and institutional barriers further hinder progress (6). A mixed-methods study highlighted that while both students and faculty viewed AI positively, significant gaps in knowledge and practical skills remained (42). Addressing these barriers through targeted AI curricula and practical training programs is crucial for enhancing students’ AI proficiency.

Unlike previous studies in developed regions, this study provides evidence from Guangxi, a less-developed area. Our findings reveal that 69.2% of participants were from rural hometowns, 70.9% had not visited a science museum or exhibition in the past year, and 66.3% had not studied AI during undergraduate education-figures likely distinct from affluent settings. Urban students had significantly higher perceived knowledge scores than rural peers, reflecting structural disadvantages (43). Positive attitudes did not automatically translate into practice when barriers prevailed-a pattern also observed in other low-resource settings (44). Perceived knowledge was strongly shaped by prior informal science exposure and formal AI education, both unevenly distributed across urban–rural lines. These findings challenge the assumption that merely adding AI courses to medical curricula will suffice in underdeveloped regions; without addressing structural inequalities, such interventions may widen the urban–rural divide. Future research should evaluate targeted interventions (e.g., mobile science outreach, open-access materials, rural teacher training) to bridge the AI education gap.

This study has several limitations. First, the study’s cross-sectional design limits the ability to infer causality between identified factors and KAP scores. Second, reliance on self-reported data may introduce biases, such as overestimation of knowledge or attitudes. Incorporating objective measures of AI knowledge and practice could enhance the robustness of the findings. Third, although the sample size was large and met statistical requirements, the use of convenience sampling instead of random sampling may affect the generalizability of the findings. Finally, the sample was skewed: 82.7% of participants were in their fifth academic year, while early-year students (years 1–4) were grossly under-represented. Consequently, the observed association that years 1–4 had higher knowledge and practice scores than fifth-year students should be interpreted with caution. Moreover, the sample was dominated by clinical medicine (56.7%) and general practice (18.5%) majors, with under-representation of other majors. While students in medical imaging appeared to have higher knowledge scores, the small sample size limits the strength of conclusions. Future studies should recruit more balanced samples across academic years and oversample non-clinical disciplines to enable robust cross-year and cross-major comparisons.

## Conclusion

Medical students in Guangxi have moderate perceived AI knowledge and positive attitudes, but structural barriers (limited practical opportunities, lack of specialized textbooks/courses, and insufficient professional guidance) hinder AI integration into medical education. Based on these findings, we propose the following actionable recommendations: (1) integrate elective AI modules and establish hands-on workshops using open-source tools to address the lack of practical opportunities; (2) develop open-access, low-cost AI learning materials tailored to resource-limited settings, and provide faculty training programs for instructors in rural-serving institutions; and (3) create cross-disciplinary collaborations with computer science departments to apply AI to real-world clinical cases. Future research should also explore structural inequalities that affect AI learning. Policy makers and medical educators should prioritize these steps to equip future doctors with essential AI competencies.

## Data Availability

The data used during the study are available from the corresponding author on reasonable requests. Requests to access these datasets should be directed to YZ, zhouyt0026@163.com.
