# Supplementary material for: Perceived knowledge, attitude, and practice of artificial intelligence among medical students in Guangxi: a cross-sectional study
Source: Front Public Health. 2026 Jun 2;14:1824962. doi: 10.3389/fpubh.2026.1824962 (PMC13269109; doi:10.3389/fpubh.2026.1824962)
Supplement: Supplementary file 1 [file Table_1.doc]

**Supplementary Table S1. The detailed expert ratings and the resulting item-level content validity indices**

| **Questionnaire** | Expert 1 | | Expert 2 | | Expert 3 | | Expert 4 | | I-CVI  (Rel) | I-CVI  (Rep) |
| --- | --- | --- | --- | --- | --- | --- | --- | --- | --- | --- |
|  | Relevance | Representativeness | Relevance | Representativeness | Relevance | Representativeness | Relevance | Representativeness |  |  |
| **Demographics section** | | | | | | | | | | |
| 1.Your academic year: | 5 | - | 5 | - | 5 | - | 5 | - | 1 | - |
| 2.Your gender: | 5 | - | 5 | - | 5 | - | 5 | - | 1 | - |
| 3.Your major: | 5 | - | 5 | - | 5 | - | 5 | - | 1 | - |
| 4.Your hometown | 5 | - | 4 | - | 5 | - | 4 | - | 1 | - |
| 5.Have you visited a science museum or exhibition in the past year? | 4 | - | 5 | - | 4 | - | 4 | - | 1 | - |
| 6.Did you study artificial intelligence during your undergraduate education? | 5 | - | 5 | - | 5 | - | 5 | - | 1 | - |
| **Knowledge section:** | | | | | | | | | | |
| 1. Do you have a solid knowledge of the basics of AI? | 5 | 5 | 5 | 5 | 5 | 5 | 5 | 5 | 1 | 1 |
| 2. Do you know what deep learning/machine learning is? | 5 | 5 | 5 | 5 | 5 | 5 | 5 | 5 | 1 | 1 |
| 3. Do you know any application of AI in your field of interest? | 5 | 4 | 4 | 4 | 4 | 5 | 4 | 5 | 1 | 1 |
| 4. AI requires a lot of labeled data to learn (data already processed by a human) | 4 | 4 | 4 | 4 | 5 | 4 | 3 | 4 | 1 | 1 |
| 5. I understand the barriers to applying AI in medicine | 4 | 4 | 4 | 3 | 5 | 5 | 5 | 4 | 1 | 0.75 |
| **Attitude section:** | | | | | | | | | | |
| 6.I believe healthcare students should learn the basics of AI | 5 | 5 | 5 | 5 | 5 | 5 | 5 | 5 | 1 | 1 |
| 7.I believe AI will be an essential tool in my field. | 5 | 5 | 5 | 4 | 5 | 5 | 5 | 5 | 1 | 1 |
| 8.I believe the ethical implications of AI must be understood by all medical students. | 5 | 5 | 5 | 5 | 5 | 5 | 5 | 5 | 1 | 1 |
| 9.I believe AI will revolutionize the educational system | 4 | 4 | 5 | 4 | 4 | 3 | 4 | 4 | 1 | 0.75 |
| 10.I believe human teachers will be replaced in the foreseeable future | 4 | 4 | 3 | 4 | 4 | 4 | 4 | 5 | 0.75 | 1 |
| 11.I believe the upcoming developments in the educational system will excite me | 4 | 4 | 4 | 4 | 3 | 4 | 4 | 4 | 0.75 | 1 |
| 12.I believe AI should be part of the training system for medical students | 5 | 5 | 5 | 5 | 5 | 5 | 5 | 5 | 1 | 1 |
| 13.I believe clinical AI will be more accurate than physicians | 4 | 3 | 4 | 4 | 4 | 4 | 3 | 4 | 0.75 | 0.75 |
| 14.I believe some specialties are more prone to be replaced by AI than others | 4 | 4 | 4 | 4 | 5 | 4 | 4 | 4 | 1 | 1 |
| 15.I believe AI would increase the percentage of errors in diagnosis | 4 | 4 | 3 | 4 | 4 | 4 | 4 | 3 | 0.75 | 0.75 |
| 16.I believe AI should be integrated into the medical curriculum | 5 | 5 | 5 | 5 | 5 | 5 | 5 | 5 | 1 | 1 |
| 17.I believe AI would promote medical development | 5 | 5 | 5 | 5 | 5 | 5 | 5 | 5 | 1 | 1 |
| **Practices section:** | | | | | | | | | | |
|  |  |  |  |  |  |  |  |  |  |  |
| 18.How frequently do you use AI to prepare for your exams? | 5 | 5 | 5 | 4 | 5 | 5 | 5 | 5 | 1 | 1 |
| 19.How frequently do you use AI to prepare for your homework/assignments? | 5 | 5 | 5 | 4 | 5 | 5 | 5 | 5 | 1 | 1 |
| 20.How frequently do you use AI to conduct your research? | 5 | 5 | 4 | 4 | 5 | 5 | 5 | 5 | 1 | 1 |
| 21.How frequently do you use AI for idea generation and brainstorming? | 5 | 4 | 5 | 4 | 5 | 5 | 5 | 4 | 1 | 1 |
| 22.How frequently do you use AI for personal choices/career guidance? | 4 | 4 | 4 | 4 | 5 | 4 | 4 | 4 | 1 | 1 |
| 23.How frequently do you use AI for spelling and grammar checking? | 5 | 5 | 5 | 5 | 5 | 5 | 5 | 5 | 1 | 1 |
| 24.How frequently do you use AI for personality development and other skills? | 4 | 4 | 4 | 4 | 5 | 4 | 4 | 4 | 1 | 1 |
| **Perspectives on AI learning:** | | | | | | | | | | |
|  |  |  |  |  |  |  |  |  |  |  |
| 1. What do you think are the learning requirements for medical students regarding AI? | 5 | 4 | 5 | 5 | 4 | 5 | 5 | 5 | 1 | 1 |
| 2. What do you think the learning methods for AI courses should be? | 5 | 4 | 4 | 4 | 5 | 3 | 5 | 4 | 1 | 0.75 |
| 3. What do you think the current learning content for AI should include? | 5 | 5 | 5 | 4 | 5 | 5 | 4 | 5 | 1 | 1 |
| 4. What difficulties do you currently face in the process of learning AI? | 4 | 4 | 5 | 4 | 5 | 5 | 5 | 5 | 1 | 1 |
| 5. What do you think are the barriers to the application of AI? | 5 | 5 | 5 | 5 | 5 | 5 | 5 | 5 | 1 | 1 |

Note: “-” Not applicable

**Supplementary Table S2. Item-Total Statistics**

| **Items** | **Scale Mean if Item Deleted** | **Scale Variance if Item Deleted** | **Corrected Item-Total Correlation** | **Cronbach’s Alpha if Item Deleted** |
| --- | --- | --- | --- | --- |
| 1. Do you have a solid knowledge of the basics of AI? | 64.60 | 89.206 | 0.450 | 0.878 |
| 2. Do you know what deep learning/machine learning is? | 64.76 | 89.118 | 0.433 | 0.878 |
| 3. Do you know any application of AI in your field of interest? | 64.54 | 88.726 | 0.479 | 0.877 |
| 4. AI requires a lot of labeled data to learn (data already processed by a human) | 64.42 | 87.479 | 0.465 | 0.878 |
| 5. I understand the barriers to applying AI in medicine | 64.61 | 88.678 | 0.476 | 0.877 |
| 6.I believe healthcare students should learn the basics of AI | 64.19 | 87.874 | 0.536 | 0.876 |
| 7.I believe AI will be an essential tool in my field. | 63.76 | 87.158 | 0.538 | 0.876 |
| 8.I believe the ethical implications of AI must be understood by all medical students. | 63.64 | 88.344 | 0.430 | 0.879 |
| 9.I believe AI will revolutionize the educational system | 64.10 | 88.346 | 0.472 | 0.877 |
| 10.I believe human teachers will be replaced in the foreseeable future | 64.85 | 90.738 | 0.255 | 0.884 |
| 11.I believe the upcoming developments in the educational system will excite me | 63.90 | 87.827 | 0.571 | 0.875 |
| 12.I believe AI should be part of the training system for medical students | 63.81 | 88.595 | 0.524 | 0.876 |
| 13.I believe clinical AI will be more accurate than physicians | 64.64 | 91.054 | 0.285 | 0.882 |
| 14.I believe some specialties are more prone to be replaced by AI than others | 63.91 | 89.623 | 0.412 | 0.879 |
| 15.I believe AI would increase the percentage of errors in diagnosis | 64.26 | 94.020 | 0.136 | 0.885 |
| 16.I believe AI should be integrated into the medical curriculum | 63.92 | 87.669 | 0.551 | 0.875 |
| 17.I believe AI would promote medical development | 63.65 | 89.023 | 0.478 | 0.877 |
| 18.How frequently do you use AI to prepare for your exams? | 64.85 | 87.125 | 0.521 | 0.876 |
| 19.How frequently do you use AI to prepare for your homework/assignments? | 64.86 | 88.044 | 0.502 | 0.877 |
| 20.How frequently do you use AI to conduct your research? | 65.15 | 87.039 | 0.536 | 0.876 |
| 21.How frequently do you use AI for idea generation and brainstorming? | 65.16 | 86.896 | 0.558 | 0.875 |
| 22.How frequently do you use AI for personal choices/career guidance? | 65.25 | 89.026 | 0.418 | 0.879 |
| 23.How frequently do you use AI for spelling and grammar checking? | 65.02 | 88.507 | 0.430 | 0.879 |
| 24.How frequently do you use AI for personality development and other skills? | 65.09 | 87.128 | 0.553 | 0.875 |

**Supplementary Table S3. Rotated component matrix**

| **Items** | **Factor 1** | **Factor 2** | **Factor 3** |
| --- | --- | --- | --- |
| 1. Do you have a solid knowledge of the basics of AI? | 0.129 | 0.099 | 0.809 |
| 2. Do you know what deep learning/machine learning is? | 0.030 | 0.138 | 0.848 |
| 3. Do you know any application of AI in your field of interest? | 0.139 | 0.113 | 0.835 |
| 4. AI requires a lot of labeled data to learn (data already processed by a human) | 0.234 | 0.055 | 0.772 |
| 5. I understand the barriers to applying AI in medicine | 0.125 | 0.124 | 0.822 |
| 6.I believe healthcare students should learn the basics of AI | 0.659 | 0.181 | 0.117 |
| 7.I believe AI will be an essential tool in my field. | 0.805 | 0.061 | 0.107 |
| 8.I believe the ethical implications of AI must be understood by all medical students. | 0.719 | -0.004 | 0.103 |
| 9.I believe AI will revolutionize the educational system | 0.682 | 0.108 | 0.031 |
| 10.I believe human teachers will be replaced in the foreseeable future | 0.151 | 0.296 | -0.008 |
| 11.I believe the upcoming developments in the educational system will excite me | 0.790 | 0.091 | 0.152 |
| 12.I believe AI should be part of the training system for medical students | 0.808 | 0.038 | 0.103 |
| 13.I believe clinical AI will be more accurate than physicians | 0.194 | 0.295 | -0.010 |
| 14.I believe some specialties are more prone to be replaced by AI than others | 0.569 | 0.133 | 0.024 |
| 15.I believe AI would increase the percentage of errors in diagnosis | 0.128 | 0.055 | 0.070 |
| 16.I believe AI should be integrated into the medical curriculum | 0.770 | 0.139 | 0.070 |
| 17.I believe AI would promote medical development | 0.773 | 0.024 | 0.095 |
| 18.How frequently do you use AI to prepare for your exams? | 0.163 | 0.753 | 0.057 |
| 19.How frequently do you use AI to prepare for your homework/assignments? | 0.094 | 0.773 | 0.088 |
| 20.How frequently do you use AI to conduct your research? | 0.025 | 0.827 | 0.187 |
| 21.How frequently do you use AI for idea generation and brainstorming? | 0.065 | 0.850 | 0.136 |
| 22.How frequently do you use AI for personal choices/career guidance? | -0.046 | 0.797 | 0.056 |
| 23.How frequently do you use AI for spelling and grammar checking? | 0.047 | 0.694 | 0.097 |
| 24.How frequently do you use AI for personality development and other skills? | 0.109 | 0.789 | 0.143 |

**Supplementary Table S4. Quantile regression (τ = 0.5) results for factors associated with medical students’ knowledge scores**

| Item | | Coefficient | Std. Error | t | *p* | 95% Confidence Interval | |
| --- | --- | --- | --- | --- | --- | --- | --- |
|  |  |  |  |  |  | Lower Bound | Upper Bound |
| (Intercept) | - | 14.000 | 0.6110 | 22.912 | <0.001 | 12.801 | 15.199 |
| Gender | Male | 1.000 | 0.2081 | 4.805 | <0.001 | 0.592 | 1.408 |
|  | Female | 0^a^ | . | . | . | . | . |
| Hometown | Urban | 1.000 | 0.2255 | 4.434 | <0.001 | 0.557 | 1.443 |
|  | Rural | 0^a^ | . | . | . | . | . |
| Have you visited a science museum or exhibition in the past year? | Yes | 1.000 | 0.2349 | 4.257 | <0.001 | 0.539 | 1.461 |
|  | No | 0^a^ | . | . | . | . | . |
| Did you study artificial intelligence during your undergraduate education? | Yes | 1.000 | 0.2242 | 4.459 | <0.001 | 0.560 | 1.440 |
|  | No | 0^a^ | . | . | . | . | . |
| Academic Year | 1-4 | 1.000 | 0.2974 | 3.362 | 0.001 | 0.416 | 1.584 |
|  | 5 | 0^a^ | . | . | . | . | . |
| Major | Clinical medicine | -2.000 | 0.6113 | -3.272 | 0.001 | -3.200 | -.800 |
|  | General practice | -2.000 | 0.6469 | -3.092 | 0.002 | -3.270 | -.730 |
|  | Other majors | -2.000 | 0.6385 | -3.132 | 0.002 | -3.253 | -.747 |
|  | Medical imaging | 0^a^ | . | . | . | . | . |
| Note: Pseudo R^2^ = 0.073. Mean Absolute Error (MAE) = 2.271812 | | | | | | | |
| a. Reference category. | | | | | | | |

**Supplementary Table S5. Quantile regression (τ = 0.5) results for factors associated with medical students’ attitude scores**

| Item | | Coefficient | Std. Error | t | *p* | 95% Confidence Interval | |
| --- | --- | --- | --- | --- | --- | --- | --- |
|  |  |  |  |  |  | Lower Bound | Upper Bound |
| (Intercept) | - | 34.750 | 1.3344 | 26.041 | <0.001 | 32.131 | 37.369 |
| Gender | Male | -0.750 | 0.3658 | -2.050 | 0.041 | -1.468 | -0.032 |
|  | Female | 0^a^ | . | . | . | . | . |
| Hometown | Urban | 0.250 | 0.3955 | 0.632 | 0.527 | -0.526 | 1.026 |
|  | Rural | 0^a^ | . | . | . | . | . |
| Have you visited a science museum or exhibition in the past year? | Yes | 1.250 | 0.4191 | 2.983 | 0.003 | 0.427 | 2.073 |
|  | No | 0^a^ | . | . | . | . | . |
| Did you study artificial intelligence during your undergraduate education? | Yes | 0.250 | 0.3980 | 0.628 | 0.530 | -0.531 | 1.031 |
|  | No | 0^a^ | . | . | . | . | . |
| Academic Year | 1-4 | 0.250 | 0.5215 | 0.479 | 0.632 | -0.774 | 1.274 |
|  | 5 | 0^a^ | . | . | . | . | . |
| Major | Clinical medicine | -1.992E-15 | 1.0739 | 0.000 | 1.000 | -2.108 | 2.108 |
|  | General practice | -1.250 | 1.1389 | -1.098 | 0.273 | -3.485 | 0.985 |
|  | Other majors | -3.536E-15 | 1.1211 | .000 | 1.000 | -2.200 | 2.200 |
|  | Medical imaging | 0^a^ | . | . | . | . | . |
| Perceived AI knowledge | - | 0.250 | 0.0578 | 4.322 | 0.000 | 0.136 | 0.364 |
| Note: Pseudo R^2^ = 0.037. Mean Absolute Error (MAE) = 3.7867 | | | | | | | |
| a. Reference category. | | | | | | | |

**Supplementary Table S6. Quantile regression (τ = 0.5) results for factors associated with medical students’ practice scores**

| Item | | Coefficient | Std. Error | t | *p* | 95% Confidence Interval | |
| --- | --- | --- | --- | --- | --- | --- | --- |
|  |  |  |  |  |  | Lower Bound | Upper Bound |
| (Intercept) | - | 7.472 | 1.4700 | 5.083 | <0.001 | 4.587 | 10.357 |
| Gender | Male | -0.833 | 0.3200 | -2.604 | 0.009 | -1.461 | -0.205 |
|  | Female | 0^a^ | . | . | . | . | . |
| Hometown | Urban | 0.111 | 0.3460 | 0.321 | 0.748 | -0.568 | 0.790 |
|  | Rural | 0^a^ | . | . | . | . | . |
| Have you visited a science museum or exhibition in the past year? | Yes | 0.361 | 0.3663 | 0.986 | 0.325 | -0.358 | 1.080 |
|  | No | 0^a^ | . | . | . | . | . |
| Did you study artificial intelligence during your undergraduate education? | Yes | 1.250 | 0.3477 | 3.595 | <0.001 | 0.568 | 1.932 |
|  | No | 0^a^ | . | . | . | . | . |
| Academic Year | 1-4 | 1.444 | 0.4556 | 3.171 | 0.002 | 0.550 | 2.339 |
|  | 5 | 0^a^ | . | . | . | . | . |
| Major | Clinical medicine | 0.056 | 0.9377 | 0.059 | 0.953 | -1.785 | 1.896 |
|  | General practice | -0.083 | 0.9943 | -0.084 | 0.933 | -2.035 | 1.868 |
|  | Other majors | 0.639 | 0.9792 | 0.652 | 0.514 | -1.283 | 2.561 |
|  | Medical imaging | 0^a^ | . | . | . | . | . |
| Perceived AI knowledge | - | 0.278 | 0.0522 | 5.319 | <0.001 | 0.175 | 0.380 |
| Attitude towards AI | - | 0.083 | 0.0285 | 2.928 | 0.003 | 0.027 | 0.139 |
| Note: Pseudo R^2^ = 0.072. Mean Absolute Error (MAE) = 3.2493 | | | | | | | |
| a. Reference category. | | | | | | | |
